# Unravelling variations: an examination of entry point selection in proximal femoral cephalomedullary nailing

**DOI:** 10.1186/s10195-024-00760-8

**Published:** 2024-04-23

**Authors:** Leonard Lisitano, Laura Wulff, Jürgen Schmidt, Christoph Sieland, Lutz Mahlke, Timon Röttinger, Jairo Cifuentes, Edgar Mayr, Kim Rau

**Affiliations:** 1 Department for Trauma, Orthopedics, Hand and Plastic Surgery, University Hospital Augsburg, Stenglinstr. 2, 86156 Augsburg, Germany; 2 Medical Care Center of Surgery and Orthopedics at Vincentinum, Augsburg, Germany; 3 Metamorphosis GmbH, Paderborn, Germany; 4 Department for Orthopedics, Trauma and Sports Traumatology, St. Vincenz-Krankenhaus, Paderborn, Germany

**Keywords:** Cephalomedullary nail, Femoral fractures, Entry point, Complications

## Abstract

**Background:**

The exact positioning of the cephalomedullary (CM) nail entry point for managing femoral fractures remains debatable, with significant implications for fracture reduction and postoperative complications. This study aimed to explore the variability in the selection of the entry point among trauma surgeons, hypothesizing potential differences and their association with surgeon experience.

**Methods:**

In this prospective multicenter study, 16 participants, ranging from residents to senior specialists, partook in a simulation wherein they determined the optimal entry point for the implantation of a proximal femoral nail antirotation (PFN-A; DePuy Synthes) in various femora. The inter- and intra-observer variability was calculated, along with comprehensive descriptive statistical analysis, to assess the variability in entry point selection and the impact of surgeon experience.

**Results:**

In this study, the mean distance from the selected entry points to the calculated mean entry point was 3.98 mm, with a smaller distance observed among surgeons with more than 500 implantations (ANOVA, *p* = 0.050). Intra-surgeon variability for identical femora averaged at 5.14 mm, showing no significant differences across various levels of surgical experience or training. Notably, 13.6% of selected entry points would not allow a proper intramedullary positioning of the implant, thereby rendering anatomical repositioning unfeasible. Among these impossible entry points, a significant skew towards anterior placement was observed (70.6% of the impossible entry points), with a smaller fraction being overly lateral (27.5%) or medial (13.7%). On a patient level, the impossibility rate varied widely from 0 to 35% among the different femora examined, with a significantly higher rate seen in younger patients (mean age 55.02 versus 60.32; *t*-test for independent samples, *p* = 0.04).

**Conclusions:**

Significant variations exist in surgeons’ selection of entry points for proximal femoral nailing, underscoring the task’s complexity. Experience does not prevent the choice of unfeasible entry points, emphasizing the inadequacy of a universal approach and pointing towards the necessity for a patient-specific strategy for improved outcomes.

*Trial registration number:* DRKS00032465.

## Key points

- The ideal entry point for cephalomedullary (CM) nailing of the proximal femur remains a matter of debate; however, there may not be a universal, perfect entry point.
- There is significant variation in the chosen entry points for CM nailing, and a substantial portion of these entry points do not allow for intramedullary positioning of the implant.

## Introduction

Pertrochanteric, subtrochanteric and femur shaft fractures represent common injuries, with increasing incidence owing to an ageing global population. The management of such fractures usually employs the insertion of cephalomedullary (CM) nails. Despite the widespread use of this approach, the exact positioning for the entry point of the CM nail, which is critical for optimal fracture reduction and the avoidance of complications, remains a matter of debate [1].

While surgical technique guides from leading manufacturers of commonly utilized nails, such as proximal femoral nail antirotation (PFN-A) and trochanteric fixation nail advanced (TFN-A) from DePuy Synthes, AUTOBAHN™ from Globus Medical and HERACLES from 7s Medical, do offer entry point recommendations, these guidelines are often general and not definitively precise. For instance, in the anterior–posterior (AP) view, the correct entry point is usually described as on the tip or slightly lateral to the tip of the greater trochanter, factoring in the nail’s medio-lateral (ML) angle [2–5]. The lateral view, however, advises an entry point centred within the trochanter, aligning with the axis of the intramedullary canal [2–5].

Studies examining the optimal entry point have yielded variable findings. One study investigating the correct entry point for subtrochanteric fractures suggested the optimal universal entry point to be at the tip of the trochanter or slightly medial to that [6]. In contrast, a more recent publication from 2020 recommended an entry point for PFN-A to be 5 mm medial to the greater trochanter tip, challenging the conventional wisdom provided by the surgical technique guide [7].

The quality of fracture reduction and nail positioning is intrinsically linked to the CM nail entry point. The defining criteria of correct nail positioning are multifaceted and complex, including factors, such as specific distances between the cortex and the nail, the central positioning of the spiral blade, avoidance of distal tip-cortex impingement and more [8–10].

Given the complexity of the criteria for correct nail positioning, this study seeks to explore the variability in entry point selection by different trauma surgeons. We hypothesize that entry point selections will differ between surgeons, and that these chosen entry points may not consistently allow for optimal fracture reduction or may cause contact between the nail and the cortical bone. We also aim to examine whether surgeon experience correlates with the consistency and quality of entry point selection.

Methods

This prospective multicenter trial was conducted at a university hospital (level I trauma centre) in collaboration with another level II trauma centre. A total of 16 surgeons, all actively engaged in trauma and orthopaedic surgery, participated in the study. The participant group comprised eight residents, three specialists and five senior specialists/attending physicians. Participant experience in terms of performed implantations varied: eight participants had performed fewer than 50 implantations themselves, four had completed between 100 and 500, two had done between 500 to 1000 and two had undertaken more than 1000 implantations.

The sample size was determined through power analysis, taking into account the expected variations of entry points. On the basis of a pre-study conducted during the development of the simulation software, more experienced surgeons were anticipated to have a mean distance to the mean entry point of 3.5 mm (SD 2.5 mm), while less experienced surgeons were expected to have a mean of 4 mm (SD 2.5 mm). This analysis determined that a minimum of 13 participating surgeons would provide the study with 80% power at an alpha level of *p* ≤ 0.05.

The trial spanned from September to December 2022, with each participant dedicating approximately 90 min to a custom-developed simulation software. All surgeons were asked to provide the optimal entry point for implantation of a PFN-A (DePuy Synthes) for different virtual femora.

The custom developed software uses digitally reconstructed radiographs (DRRs) to provide X-ray views from computed tomography (CT)-datasets. To simulate these images, a method called ‘ray casting’ is employed. This technique involves casting rays from a virtual focal point towards an image plane, capturing data along the ray’s path via trilinear interpolation [11]. The attenuation of each pixel is computed from the CT data and then transformed into an 8-bit grayscale image, with certain adjustments to enhance realism and contrast. DRRs are all but indistinguishable from actual X-rays (voxel size: coronary 1.02 mm, sagittal 1.02 mm axial 0.55 mm).

**Fig. 1:**
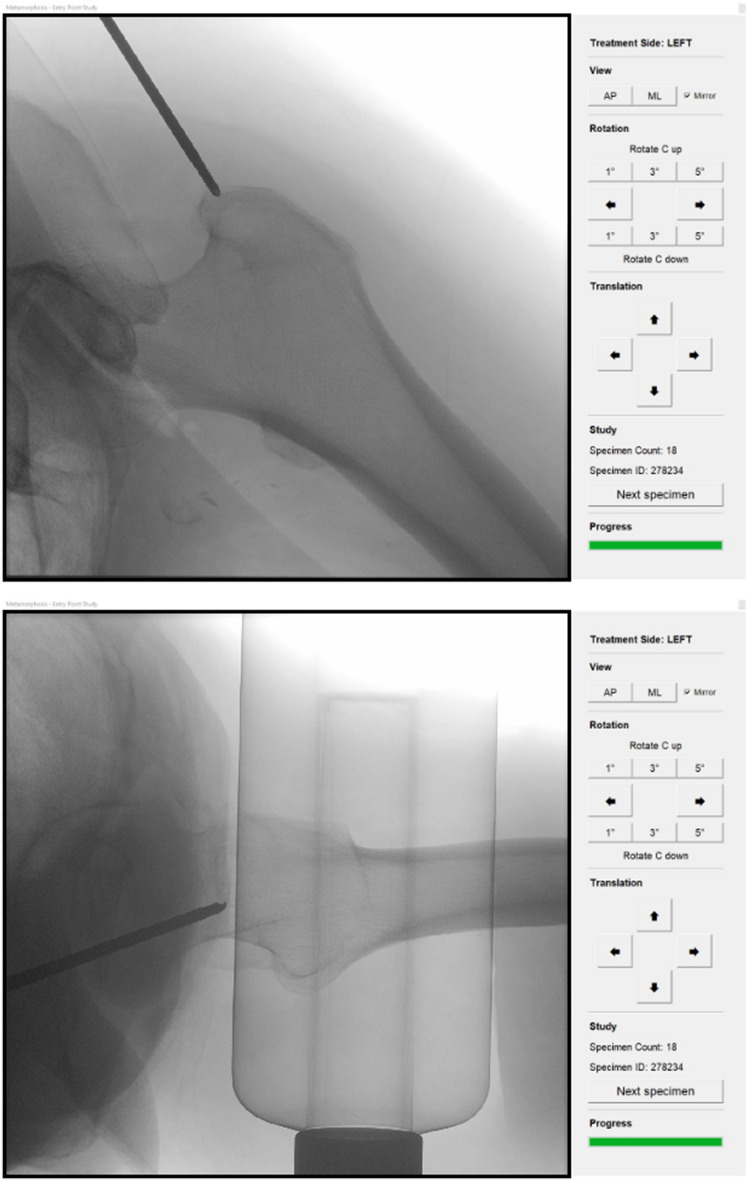
Simulation software interface. This figure illustrates the user interface of the simulation software, showcasing a realistic femur image. Participants determine the optimal implant entry point by clicking directly on the image. The control panel on the right allows image manipulation, such as rotation and zooming, providing a realistic surgical experience for evaluating entry point variability

Utilizing this method, the software displays two-dimensional images akin to those provided by a fluoroscopy machine. The surgeons could manipulate these images in all dimensions, just as they would during actual surgery. The guiding wire was visible in all images to replicate reality, and the simulated fluoroscopy machine could be adjusted by 0.5 °. Surgeons could always switch between the AP and ML view to ensure that the entry point was determined from at least two views. The guiding wire could be repositioned as needed until the participant confirmed the final position. The participant’s view is shown in Fig. 1.

For the entirety of the analysis, the PFN-A by DePuy Synthes was utilized as it was the primary implant employed at the participating hospitals.

Additionally, the simulation software established a randomized pattern. This was achieved by autonomously selecting the order and orientation of the 19 proximal femora, each presented to the participants at least three times in a mirrored or unmirrored form. In total each surgeon provided at least 57 entry points. This methodology allowed for the examination of whether the same surgeon would consistently select the same entry point on identical femora. The distance between the chosen entry points for each femur was subsequently calculated.

The mean entry point for each femur was calculated from the 90% closest entry points determined by all surgeons, and the distance from each individual entry point to this mean point was measured at the bone surface (definition of mean entry point). This enabled a comparison of entry point variability and facilitated an investigation into potential differences owing to the experience level and training stage of the surgeons.

Importantly, certain entry points were deemed unsuitable for achieving an anatomical reduction. These are entry points where the distance between the surface of the implant and the surface of the bone was less than 1 mm in some place, which makes an intramedullary position of the implant impossible (negative values indicated an implant position outside the bone). Consequently, in the event of a fracture, an anatomical or near-anatomical reduction is impossible using these entry points. The software was used to calculate the implant’s position relative to the bone, thereby identifying these unsuitable entry points.

Confidentiality was preserved by only documenting the stage of surgical training and experience of each participant without including any identifying information. All participants granted their written consent for publication.

The demographic profile of the used CT datasets is characterized by a mean age of 59.37 ± 20.84 years, with a range extending from a minimum age of 22 to a maximum of 85 years. The sample includes a total of 13 male participants and 6 female participants.

This study was conducted in strict adherence to the Declaration of Helsinki and all of its amendments, ensuring the ethical conduct of research involving human subjects. Prior to its commencement, the study received the necessary approval from the ethics committee of Ludwig-Maximilians-University of Munich.

The statistical analysis primarily involved the calculation of inter-observer and intra-observer variability to assess the variability of entry points among the surgeons and between the entry points provided by the same surgeon. In addition, a comprehensive descriptive statistical analysis was conducted, encapsulating data on the chosen entry point, the surgeon’s experience level and training stage. The statistical significance was tested using Fisher’s exact test, *t*-test for independent samples and analysis of variance (ANOVA).

## Results

**Fig. 2:**
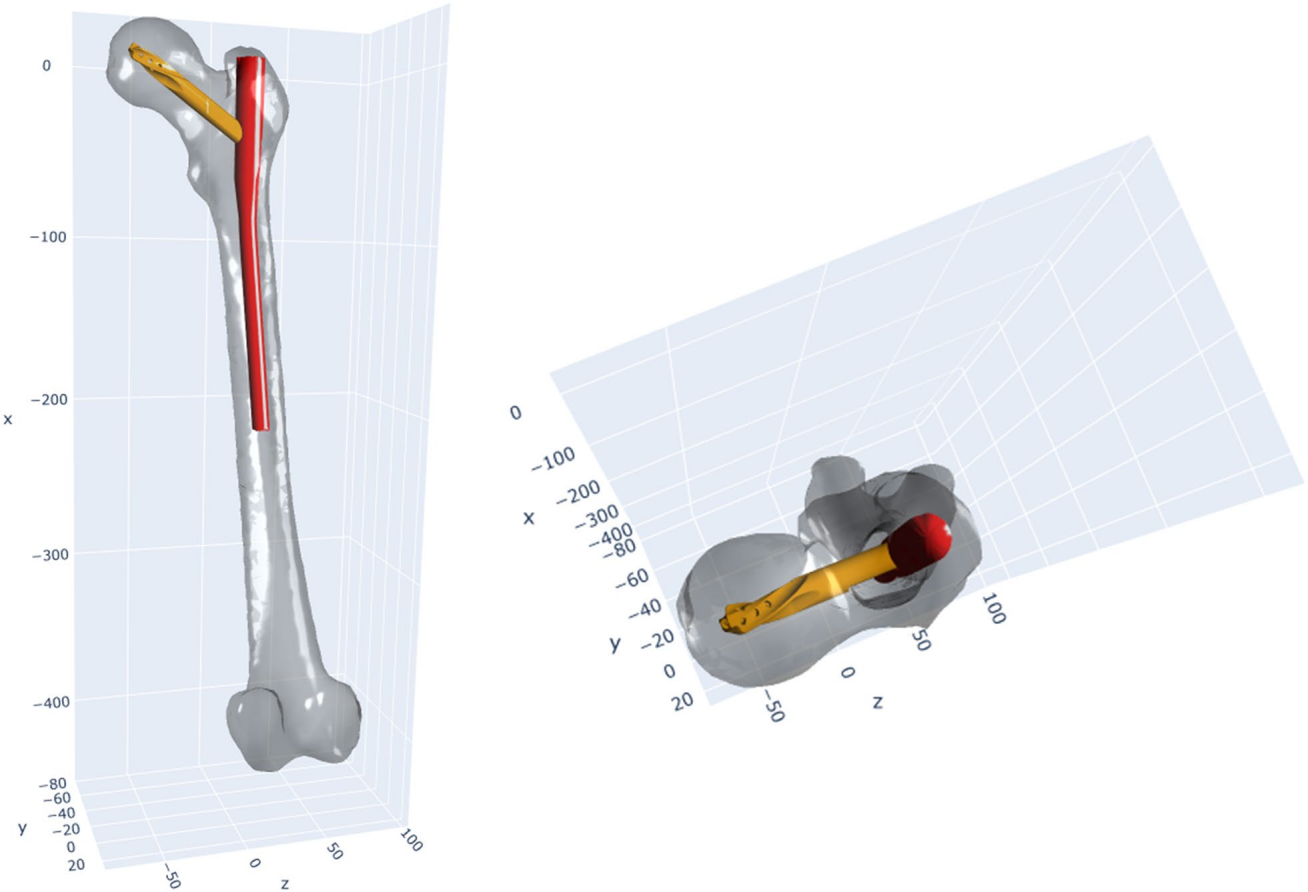
Representative PFN-A placement. These figures exemplify the placement of a PFN-A in a femur, guided by the mean entry point identified in this study. The images display an optimal intramedullary positioning of the implant, with the blade positioned centrally, attesting to the successful realization of the intended centre–centre positioning

In the overall analysis, the distances to the mean entry point exhibited a mean of 3.98 mm ± 1.81 mm. All mean entry points resulted in a satisfactory nail placement, allowing for a possible intramedullary position of the implant (Fig. 2). The recorded distances spanned a range from a minimum of 1.17 mm to a maximum of 15.49 mm. Surgeons who had completed more than 500 implantations demonstrated significantly lower distances to the mean entry point when compared with surgeons with fewer than 500 implantations (*T*-test, *p* = 0.05).

**Fig. 3:**
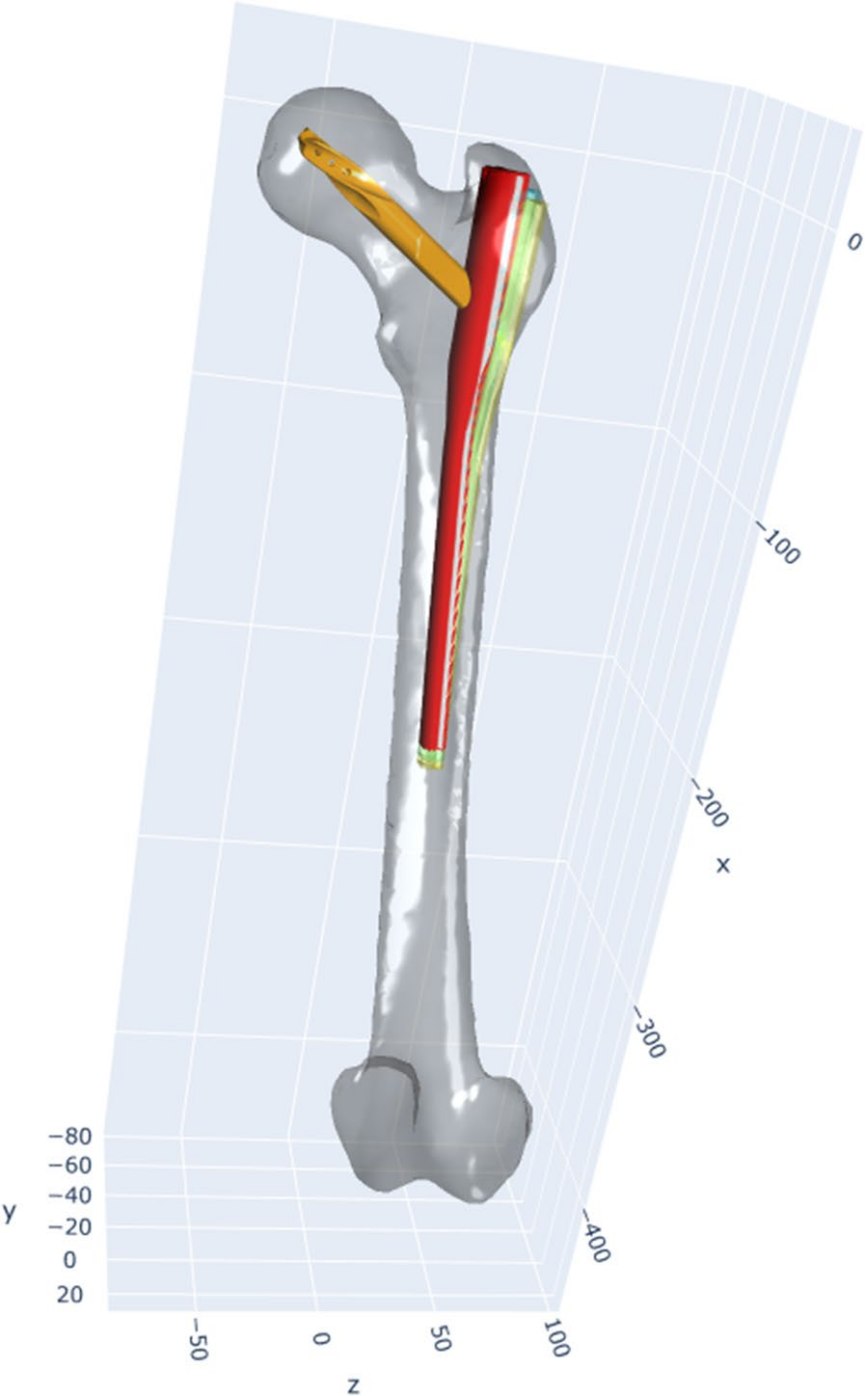
PFN-A placement for different entry points and displays the varying positions of a PFN-A, inserted at the mean entry point (red), and those inserted 5 mm (blue) and 10 mm (yellow) away from the mean entry point

**Fig. 4:**
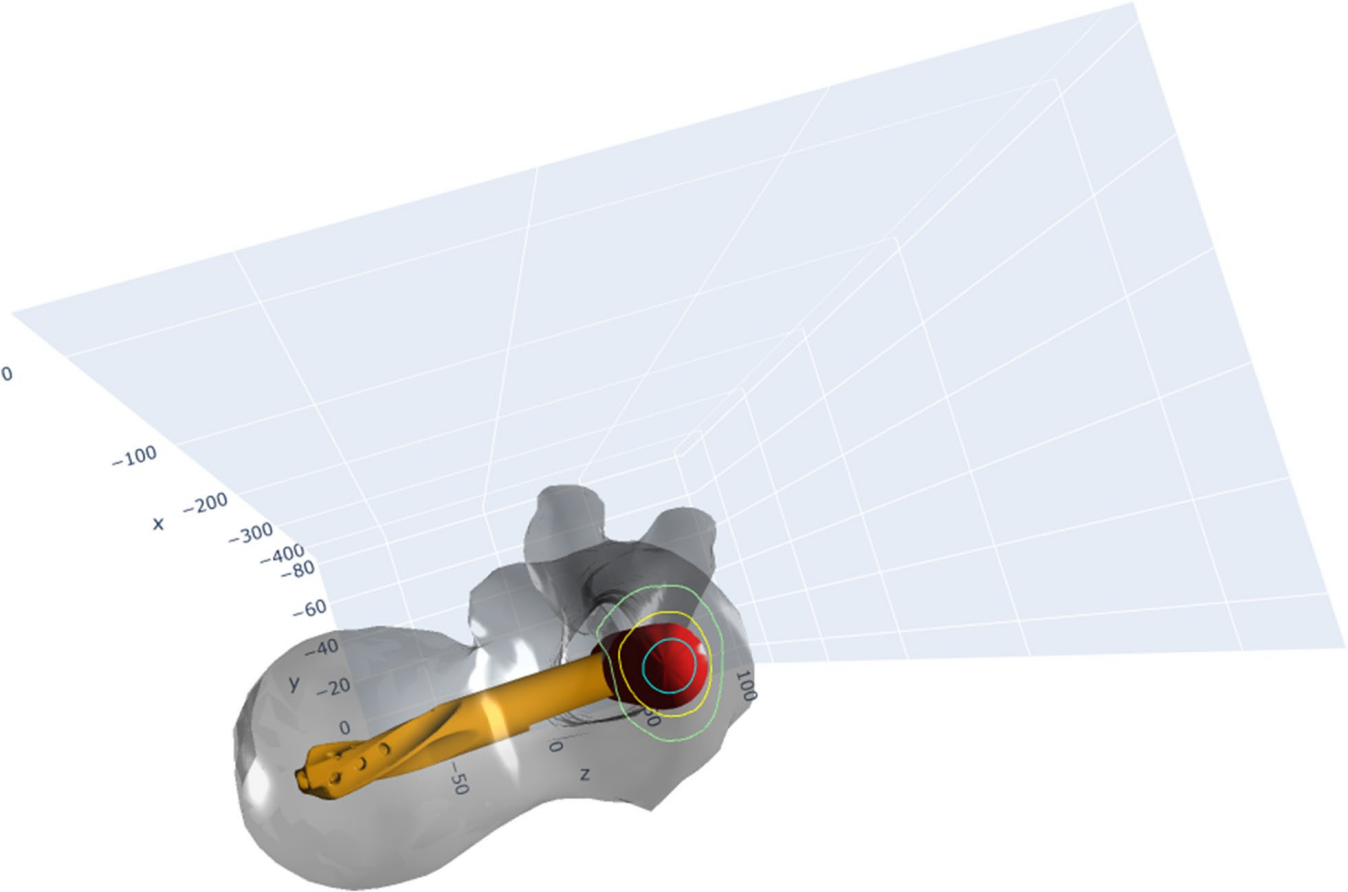
Surface lines on the bone, situated 5 mm, 10 mm and 15 mm around the mean entry point, emphasizing the differences in PFN-A positioning with various entry points

When focusing on the distances between entry points chosen by the same surgeons on identical femora, a mean distance of 5.14 mm ± 2.65 mm was observed. The smallest recorded distance was 0.56 mm, while the largest stretched to 18.4 mm. A trend towards smaller mean distances was observed among more experienced surgeons and senior specialists (Table 1). However, when assessed for statistical significance, no significant differences could be identified in relation to the varying levels of experience or stages of training among the surgeons. The effect of different entry points on the final PFN-A position is demonstrated in Figs. 3 and 4.

**Table 1:** Distance between different entry points at identical femura

	Distance between entry points
Residents	5.35 mm ± 3.01 mm
Specialists	5.27 mm ± 1.79 mm
Senior specialists	4.52 mm ± 2.02 mm

With regards to the selection of impossible entry points, as defined in the methods section, a total of 13.6% (102 out of 748) of the entry points rendered an intramedullary positioning of the implant, and subsequently, an anatomical repositioning in the case of a fracture, unattainable. Upon analysis, no significant difference was observed among the various levels of experience, categorized as less than 50 implants, between 50 and 500 implants and over 500 implants. Statistical assessment through ANOVA yielded a *p*-value of 0.065, with a maximum observed average value of 18.0% and a minimum of 11.2% for the different groups.

**Fig. 5:**
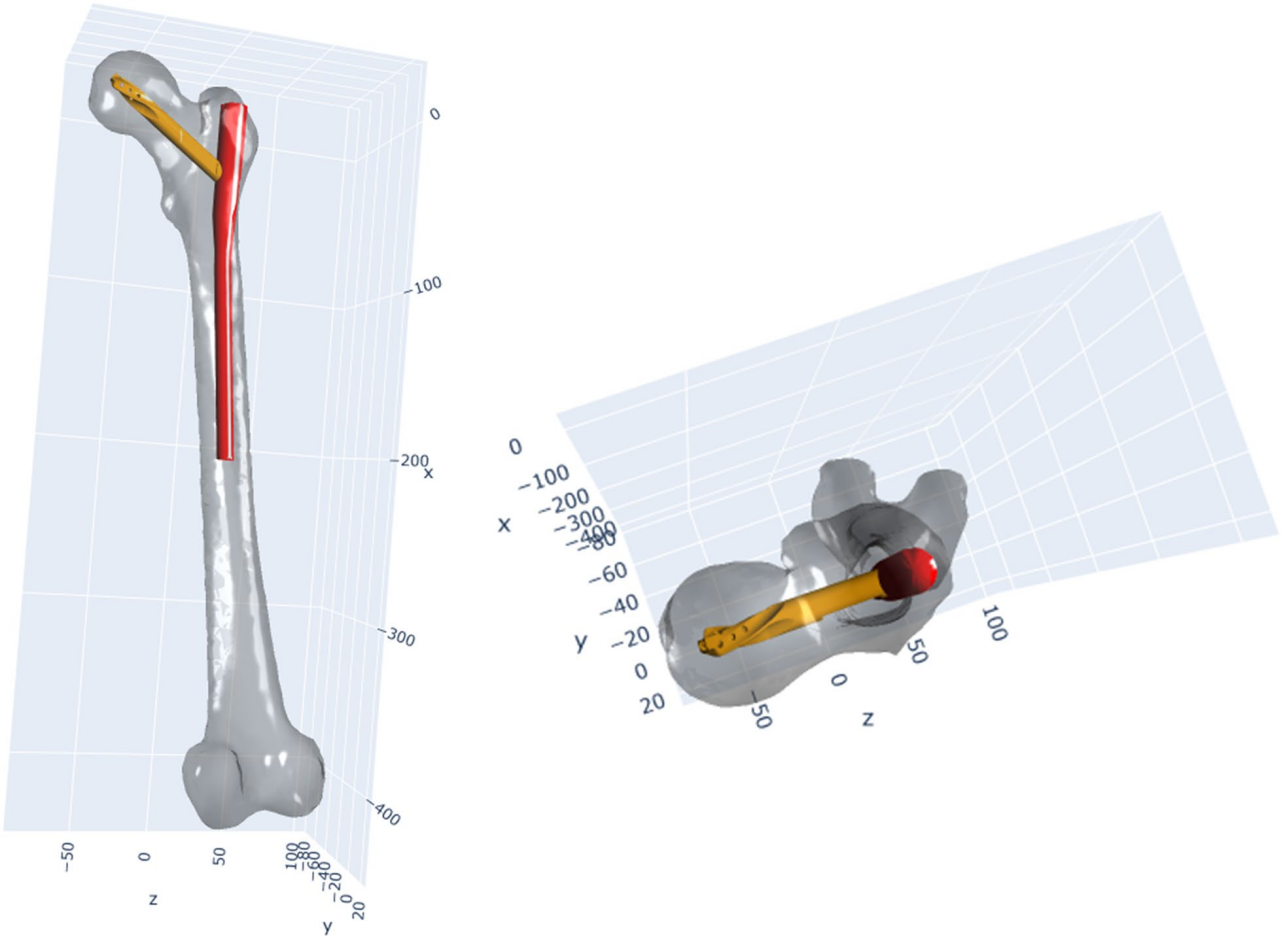
Overly lateral entry point PFN-A placement. These figures demonstrate the placement of a PFN-A using an overly lateral entry point. In this scenario, the implant partially resides in the cortical bone or even outside the bone structure. If a fracture were present, this entry point would not allow for an accurate anatomical repositioning. Consequently, the implant could not be inserted without complications

In the analysis of the directionality of the 102 identified impossible entry points, a predominant skew toward anterior placement was observed. Of these, a total of 72 were excessively anterior (52 purely anterior, 13 both anterior and lateral and 7 both anterior and medial). Only three were classified as overly posterior. Furthermore, 28 entry points were classified as unachievable owing to excessive lateral placement (13 were solely lateral, 13 both lateral and anterior and 2 lateral and posterior), while 14 entry points were excessively medial (6 solely medial, 7 medial and anterior and 1 medial and posterior). Figure 5 show an example for an overly lateral placed entry point.

For the femora examined in this study, the percentage of impossible entry points ranged widely from 0 to 35%, depending on the specific bone. Notably, within the group classified as impossible entry points, the patients were significantly younger than those within the possible entry points group (mean age 55.02 versus 60.32; *t*-test for independent samples, *p* = 0.04). No significant differences were found with regards to sex.

## Discussion

Although this study indicates that more experienced surgeons typically select entry points significantly closer to the mean entry point, no significant difference was found concerning the selection of impossible entry points among different experience levels. In fact, none of the groups achieved a satisfactory level of good/possible entry point selection. Furthermore, even with heightened experience, there persists an inherent variability in entry point selection, emphasizing the intricate challenge of determining the optimal nailing entry point in proximal femoral fractures.

The epidemiology of patients providing CT data for this study encompasses a broad age range, which ensures coverage of the diverse morphologies of the trochanteric region. Consequently, the demographic does not directly align with the typical patient population suffering from proximal femur fractures. The notable number of infeasible entry points observed, even among experienced surgeons, may be attributed to their familiarity with the anatomy of the elderly, predominantly female patients who typically present with these fractures. This observation is further corroborated by the finding that patients with infeasible entry points were significantly younger than those without.The results align with numerous studies examining optimal entry points for proximal femur nailing. Some studies suggest an entry point medial to the greater trochanter’s tip even when using valgus bend nails [12]. However, other research indicates a wide range of ideal entry points for different femoral configurations, including some at the tip of the greater trochanter to even more lateral positions (for varus bend nails) [6, 13]. Although, it is known that excessively lateral entry points can lead to a varus displacement, subsequently increasing the risk of complications [14]. Thus, precise entry point selection is critical in successful proximal femur nailing procedures.

The present study notably found that the majority of unfeasible entry points were excessively anterior or lateral. This observation supports existing literature advocating for a more medial entry point in proximal femur nailing procedures [12]. Complementarily, another study revealed that the optimal entry point in the sagittal plane is consistently slightly posterior to the axis of the femoral neck [15]. This finding resonates with the present study’s identified unfeasible entry points predominantly located in anterior positions. Moreover, the optimal entry point has been demonstrated to vary in accordance with the neck-shaft angle; as this angle increases, a more medial entry point is warranted [15].

Effective femoral nailing necessitates meticulous determination of the entry point, hinging on two critical parameters: alignment with the femoral shaft axis and precise ‘centre–centre’ blade positioning in the femoral head. Firstly, aligning the intramedullary nail with the femoral shaft axis is imperative for preserving the limb’s mechanical axis, thus mitigating risks of malunion and non-union [16]. Secondly, the blade’s placement in the femoral head warrants careful attention, with the ‘centre–centre’ positioning in both coronal and sagittal planes being optimal. Correct blade positioning minimizes implant failure risks and promotes favourable biomechanical outcomes.

Several anatomical studies have demonstrated significant variability in the relationship between the greater trochanter and the femoral shaft [13, 17–19]. In consideration of the differences described in the ‘optimal’ entry points, this variability presents two central challenges: firstly, it implies that a single universal entry point may not exist; secondly, it casts doubt on the reliability of anatomical landmarks as a method for identifying the correct entry point of a CM nail.

With this understanding that a single universal entry point may not exist, it becomes imperative to determine an optimal entry point for each patient individually [13]. This introduces the next challenge: once the optimal entry point is determined, it must be accurately located intraoperatively. In this context, the role of future technology becomes evident. We envision the employment of artificial intelligence (AI)-supported navigation in future procedures, potentially providing invaluable assistance, especially to less experienced surgeons, in accurately finding the determined optimal entry point.

To our knowledge, this is the first study to compare the actual entry points for PFN-A through a simulation among various surgeons. One of the strengths of our study is the randomized presentation of femora in a highly realistic simulation, which allowed for excellent comparability of results. Moreover, our approach enabled the evaluation of entry points chosen by different surgeons on the same femur, providing unique insights into the variability of entry point selection across various surgical experiences.

Despite its strengths, our study is not without limitations. While the simulation provides highly realistic images, it remains a simulation. In particular, the tactile sense of the guide wire’s location relative to the bone, which surgeons usually assess with their fingers, could not be reproduced in the simulation. Therefore, participants had to rely entirely on fluoroscopy. Nevertheless, we believe that the simulation environment yielded meaningful and reliable results, thus offering valuable insights into the selection of entry points at the proximal femur.

## Conclusions

The present study illuminates considerable variations in the determination of proximal femoral nailing entry points among different surgeons, underpinning the inherent complexity of the task. Notably, even with increasing experience levels, surgeons did not avoid choosing ‘impossible’ entry points. These findings clearly demonstrate that a ‘one-entry-point-fits-all’ approach may not be applicable in selecting the entry point owing to substantial anatomical variations among patients. Thus, a tailored approach to define an individualized optimal entry point for each patient could be the way forward in improving the outcomes of proximal femoral fracture management.

## Data Availability

The datasets used and analysed during the current study are available from the corresponding author on reasonable request.

## Abbreviations

AI: Artificial intelligence
AP: Anterior–posterior
CM nail: Cephalomedullary nail
DRR: Digitally reconstructed radiograph
ML: Medio-lateral
